# Copper-Based Nanomedicines for Cuproptosis-Mediated Effective Cancer Treatment

**DOI:** 10.34133/bmr.0094

**Published:** 2024-10-18

**Authors:** Dahye Noh, Hokyung Lee, Sangmin Lee, In-Cheol Sun, Hong Yeol Yoon

**Affiliations:** ^1^ Medicinal Materials Research Center, Biomedical Research Institute, Korea Institute of Science and Technology (KIST), Hwarang-ro 14-gil 5, Seongbuk-gu, Seoul 02792, Republic of Korea.; ^2^ Division of Bio-Medical Science & Technology, KIST School, University of Science and Technology (UST), Hwarang-ro14-gil 5, Seongbuk-gu, Seoul 02792, Republic of Korea.; ^3^ Department of Fundamental Pharmaceutical Sciences, College of Pharmacy, Kyung Hee University, 1 Hoegi-dong, Dongdaemun-gu, Seoul 02447, Republic of Korea.

## Abstract

The recent discovery of cuproptosis, a novel copper-ion-induced cell death pathway, has suggested the novel therapeutic potential for treating heterogeneous and drug-resistant cancers. Currently, copper ionophore-based therapeutics have been designed to treat cancers, utilizing copper ions as a strategic tool to impede tumor proliferation and promote cellular demise. However, limitations of copper ionophore-based therapies include nontargeted delivery of copper ions, low tumor accumulation, and short half-life. Strategies to enhance specificity involve targeting intracellular cuproptosis mechanisms using nanotechnology-based drugs. Additionally, the importance of exploring combination therapies cannot be overstated, as they are a key strategy in improving the efficacy of cancer treatments. Recent studies have reported the anticancer effects of nanomedicines that can induce cuproptosis of cancer both in vitro and in vivo. These cuproptosis-targeted nanomedicines could improve delivery efficiency with the pharmacokinetic properties of copper ion, resulting in increasing cuproptosis-based anticancer effects. This review will summarize the intricate nexus between copper ion and carcinogenesis, examining the pivotal roles of copper homeostasis and its dysregulation in cancer progression and fatality. Furthermore, we will introduce the latest advances in cuproptosis-targeted nanomedicines for cancer treatment. Finally, the challenges in cuproptosis-based nanomedicines will be discussed for future development directions.

## Introduction

Copper ion is an essential cofactor for numerous enzymes and plays a crucial role in various metabolic processes [1]. Additionally, it acts as a vital component in cytochrome c oxidase, also referred to as complex IV, within the mitochondrial electron transport chain (ETC), thereby regulating cell growth and function through efficient energy conversion [2]. Inadequate copper ion levels can impede growth, while excessive exposure to copper ion can induce oxidative stress, leading to cell death and tissue damage [3]. Thus, it has been shown that disruptions in copper homeostasis can result in structural abnormalities or the impairment of essential physiological functions [4]. Notably, variations in copper ion levels have been observed in many cancer cells, associated with elevated intratumoral copper ion concentrations and shifts in systemic copper ion distribution [5]. These alterations in copper homeostasis can adversely affect biological function and contribute to angiogenesis, chemoresistance, immune evasion, and metastasis, particularly in cancers [6].

Recent findings suggest that copper ion plays a central role in determining the proliferation (cuproplasia) and cell death (cuproptosis) [7,8]. While copper ions act as indispensable cofactor for numerous enzymes and are pivotal in various metabolic processes, disturbances in copper ion homeostasis can have detrimental effects on biological function [1,6]. A schematic illustration of the biological roles of copper ions is shown in (Fig. S1). Indeed, the proliferation and metastasis of cancer cells are widely recognized to be significantly more dependent on copper ion than normal tissues [5]. Therefore, a novel form of copper-ion-induced cell death, cuproptosis, has recently been utilized for anticancer drug discovery due to the potential for alternative pathways beyond programmed cell death mechanisms [8,9]. Cuproptosis is triggered by excess copper ions. High levels of copper ion within cells lead to the direct binding of lipoylated proteins involved in glycolytic metabolism, disrupting the tricarboxylic acid (TCA) cycle. This disruption causes aggregation of lipoylated proteins, resulting in the loss of iron-sulfur (Fe-S) clusters. Subsequently, these processes promote proteotoxic stress, ultimately leading to cell death [8]. Given its contribution to cancer proliferation, angiogenesis, and metastasis, copper-ion-induced cuproptosis has been considered to hold great potential for cancer treatment. Furthermore, the anticancer drug, disulfiram (DSF), is a clinically approved copper ionophore, facilitating the redistribution of copper ion stores across cellular and subcellular compartments [10–14]. Over the past several years, various copper-related signal pathways have been explored. As a result, cuproptosis induced by copper-based therapeutics has been refocused for cancer therapy due to its unique cell death mechanism, contrasting with apoptosis triggered by conventional anticancer agents [15,16]. Nevertheless, cuproptosis-based cancer treatment has several limitations, including the selective increase of copper ion concentration in cancer cells, prevention of copper ion damage to normal cells, and extension of cuproptosis duration [17–19]. Nanomedicine provides an effective treatment approach by leveraging nanoparticles to enhance drug solubility, extends drug circulation in the bloodstream, and regulates drug release, enhancing in vivo therapeutic efficacy and reducing off-target effects [20]. Copper-based nanomaterials offer an innovative option for cupoptosis by facilitating precise targeting and accumulation at tumor sites through the enhanced permeability and retention effect [21,22]. Furthermore, various combination approaches using cuproptosis-targeted nanomedicines and other therapeutic options, including photodynamic therapy, immunotherapy, gene therapy, and chemotherapy, enhance therapeutic outcomes. This review will introduce copper metabolism with dysregulation in cancer cells and recent advances in cuproptosis-targeted nanomedicines for effective cancer treatment. Finally, the future perspectives of cuproptosis-targeted nanomedicines will be discussed for research and clinical applications.

## Copper Homeostasis and Dysregulation in Cancer Cells

Copper ions are one of the critical trace elements essential for various physiological functions within the body [23]. It exists in 2 distinct ionic forms: Cu^+^ (cuprous ion, reduced form) and Cu^2+^ (cupric ion, oxidized form), both actively engaging in the enzymatic regulation of various cellular physiological processes, including gene expression, biological metabolic processes, mitochondrial respiration, and antioxidation [24,25]. The conversion between Cu^+^ and Cu^2+^ gives copper ion unique redox properties, making it a powerful reactive oxygen species (ROS) producer and important for ROS scavenging [26,27]. Cancer cells often have higher levels of ROS than normal cells due to several factors, such as increased metabolic activity, gene mutations, and relative hypoxia [28–30]. This imbalance between oxidants and antioxidants can contribute to the promotion of tumorigenesis and cancer progression [31,32]. Furthermore, cancer cells often exhibit a heightened dependency on copper ion due to its vital roles in various cellular processes that support tumor growth and progression [4]. Elevated copper ion levels in cancer cells can regulate angiogenesis, metastasis, and resistance to cell death pathways [33–35]. Copper ion activates enzymes and signaling pathways that promote these processes, making it a crucial element for sustaining the malignant phenotype of cancer cells [36]. This section introduces copper metabolism and homeostasis, examining how copper dysregulation contributes to cancer cells.

### Systemic copper metabolism and homeostasis

Copper ion is absorbed from dietary sources primarily by the small intestine. This process involves the uptake of copper ion by the Cu transport protein 1 (CTR1) or solute carrier family 31 member 1 (SLC31A1) across the apical membrane of enterocytes. Once inside the enterocyte, copper ion is transported across the cell and exported into the interstitial fluid and bloodstream by a protein called ATPase copper-transporting alpha/beta (ATP7A/B) located on the basolateral membrane [7,37]. Extracellular copper ions typically exist in the oxidized form, Cu^2+^. However, Cu^2+^ cannot directly enter cells due to its charge and size. To facilitate cellular uptake, Cu^2+^ is reduced to Cu^+^ by metalloreductases, such as 6-transmembrane epithelial antigen of the prostate or other reductases on the cell membrane [38].

The processes and key regulators of copper ion metabolism are summarized in (Fig. S2). Upon entry into cells, copper ion interacts with a diverse array of copper chaperone proteins, such as antioxidant 1 copper chaperone (ATOX1), copper chaperone for superoxide dismutase, and superoxide dismutase 1 (SOD1), facilitating its delivery to distinct subcellular compartments, including mitochondria, the trans-Golgi network, and the nucleus [39]. In the mitochondria, copper ion can bind to cytochrome C oxidase and contribute to the respiratory chain and redox pathways [40]. In the nucleus, copper ion can regulate gene expression by engaging with several transcription factors [41]. Once copper ion enters the circulatory system, it binds to various plasma proteins for transport to organs and tissues throughout the body. Ceruloplasmin is one of the primary copper ion-binding proteins in plasma and plays a crucial role in copper ion transport and homeostasis. Copper ion can bind to other plasma proteins such as albumin, trans copper protein, and metallothionein, which help facilitate its distribution to various target tissues and organs [42–44]. The absorbed copper ion is mainly transported to the liver, where hepatocytes are the major storehouse [45]. The liver also acts as the primary site for copper ion removal through hepatobiliary excretion via the copper ion exporter ATPase copper ion transporting beta (ATP7B) across the bile canalicular membrane of hepatocytes [45–47]. Alternative pathways for copper ion elimination, such as urine, sweat, and menses, have minimal impact on copper loss.

The regulation of copper homeostasis relies on the intricate coordination of its intake, transport, and clearance, which are pivotal in governing physiological processes [48]. In the cytoplasm, copper ion levels are regulated by various proteins, such as cuproenzymes, copper chaperones, and membrane transporters [5]. These proteins are essential for avoiding the adverse consequences of copper ion deficiency and copper ion overload, working together to maintain a precise balance of copper ion concentrations within cells [49]. They also exhibit a high affinity for copper ion binding and facilitate practically irreversible metal transfer among cognate molecules [50–52].

Reduced copper ion concentrations have been associated with conditions such as albinism, osteoporosis, and other diseases [53]. Furthermore, a deficiency of copper ion can affect brain development, highlighting the vital role of copper ion in the body [54–56]. Maintaining copper ion homeostasis is essential for cellular functions and is regulated by a network of proteins that facilitate copper ion uptake, distribution, and elimination. This is crucial for overall physiological processes and prevents adverse health outcomes associated with copper ion deficiency or overload.

### Copper ion dysregulation in cancer

The association between copper ion and cancer has been established over many years, with studies consistently showing elevated levels of copper ion and ceruloplasmin in cancers or the serum of animal models and cancer patients [7,57–60]. Copper ion is an essential cofactor for enzymes involved in vital cellular processes such as mitochondrial respiration, antioxidant defense, hormone synthesis, neurotransmitters, and pigments. However, disturbances in copper ion homeostasis can lead to oxidative stress and cytotoxicity [61,62]. Recent studies have revealed elevated levels of copper ion in the serum or tissues of patients with various malignancies, including colorectal cancer, gallbladder cancer, and thyroid cancers [63–66]. Furthermore, cancers often require elevated levels of copper ions compared to surrounding healthy tissues to support angiogenesis, growth, metastasis, and resistance to cell death [6,67].

#### Angiogenesis

Angiogenesis, the process by which new blood vessels sprout from existing blood vessels, especially capillaries, is essential for various physiological processes [68]. In normal physiology, angiogenesis ensures proper blood supply to tissues and organs, allowing for their growth, repair, and metabolic needs [69]. However, intussusceptive angiogenesis has been implicated in numerous cancer types, including melanoma, colorectal, glioma, and mammary cancers [70–73]. By stimulating the formation of new blood vessels, cancers ensure a steady supply of oxygen and nutrients, allowing them to grow and metastasize [74,75].

Copper ion is closely associated with promoting angiogenesis [76]. Gérard et al. [77] reported that copper ion promotes endothelial cell proliferation and enhances angiogenesis in vitro and in vivo. Specifically, copper ion reduced the risk of ischemia in skin flaps and induced the formation of a vascularized capsule around a cross-linked hyaluronic acid (HA)-composed hydrogel [78–81]. It has been demonstrated that copper ion plays critical roles in angiogenesis and metastasis by stimulating the production of several proangiogenic factors, including interleukin-1 (IL-1), IL-6, IL-8, tumor necrosis factor-alpha, angiogenin, vascular endothelial growth factor (VEGF) [82], SOD1 [83], hypoxia-inducible factor-1-alpha (HIF-1α) [84], and fibronectin [85,86].

In other instances, Ungar-Waron et al. [87] reported on serum ceruloplasmin levels in rabbits during tumor development and regression. Ceruloplasmin levels, the primary copper ion binder in serum, rose significantly during tumor progression and returned to normal during regression but remained elevated during metastasis. Moreover, cancer angiogenesis is necessary for tumor growth progression, wherein copper ion is involved in tumor neovascularization by directly interacting with angiogenic factors (VEGF and fibroblast growth factor) [79,88]. Upon VEGF stimulation, CTR1 undergoes rapid sulfenylation at its cytoplasmic C-terminal Cys189. This event forms a disulfide bond between CTR1 and VEGFR2, leading to their cointernalization with early endosomes, thereby sustaining VEGFR2 signaling. Mice with endothelial cell-specific CTR1 deficiency or mutations exhibit impaired developmental and reparative angiogenesis in vivo [89]. In other words, the binding of copper ion with angiogenin [79] suggests that copper ion-activated angiogenin may interact more efficiently with endothelial cells, potentially enhancing its capacity to promote the formation of new blood vessels [80]. Additionally, vascular copper ion transport systems profoundly influence the activation and execution of angiogenesis, serving as multifunctional regulators of distinct proangiogenic pathways [81]. The involvement of copper ion may represent a critical mechanism in regulating the formation of new blood vessel pathways, providing valuable insights for developing innovative therapies for cancer treatment.

#### Drug resistance

Drug resistance represents a significant challenge in cancer treatment, impacting a large number of patients with metastatic cancer [90]. It can manifest through multiple mechanisms, including limiting the uptake of drugs, altering drug targets, inactivating drugs, and actively pumping out drugs [91]. These mechanisms, among others, neutralize chemotherapy, resulting in treatment failure and disease progression [92,93]. Emerging evidence suggests that copper ion transport mechanisms could be implicated in drug resistance [94]. Majumder et al. [95] investigated the correlation between copper ion levels and drug resistance to identify patients resistant to treatment, aiming to develop improved therapeutic strategies. They revealed that blood serum from tumor-bearing mice displayed increased copper ion levels compared to healthy normal mice. Moreover, doxorubicin-resistant Ehrlich ascites carcinoma- or cyclophosphamide-resistant Lewis lung carcinoma-bearing mice exhibited higher copper ion levels in the serum compared to those of drug-sensitive tumor-bearing mice. Furthermore, the analysis of copper ion levels in healthy volunteers and cancer patients indicated a correlation between copper ion, tumor growth, and drug resistance. In addition, Jin et al. [96] investigated how elevated copper ion levels partially contribute to drug resistance and the repair of damaged DNA in cancer cells. ATOX1-induced expression of mediator of DNA damage checkpoint 1 (MDC1), a crucial protein involved in double-strand DNA damage repair. Specifically, ATOX1, acting as a copper ion chaperone, is translocated to the nucleus to target the MDC1 promoter after exposure to various genotoxic agents, promoting the transcription of MDC1 in a copper ion-dependent manner. Consequently, knockout or blockade of ATOX1 rendered cancers sensitive to gemcitabine in transplanted cancer mouse models. These observations suggest that modulating copper ion levels may improve the effectiveness of cancer chemotherapy in drug-resistant patients. By targeting the elevated copper ion levels associated with drug resistance, it may be possible to enhance the sensitivity of cancer cells to chemotherapy, thereby improving treatment outcomes. This approach could lead to the development of new therapeutic strategies aimed at overcoming drug resistance in cancer patients.

#### Immune evasion

Programmed cell death-ligand-1 (PD-L1) expressed in cancers is believed to suppress tumor-infiltrating lymphocytes via programmed cell death-1, thereby facilitating adaptive immune resistance [97]. Interestingly, copper ion contributes to these immune responses [98]. Voli et al. [15] demonstrated a correlation between intratumoral copper ion levels and PD-L1 expression in cancer cells. Copper ion supplementation boosted PD-L1 expression at both mRNA and protein levels in cancer cells. Furthermore, RNA sequencing unveiled that copper ion regulates critical signaling pathways responsible for PD-L1-mediated cancer immune evasion. An in-depth analysis of The Cancer Genome Atlas database and tissue microarrays revealed a robust association between the CTR1 and PD-L1 expression across multiple cancers, contrasting with the lack of correlation in corresponding normal tissues. In addition, Zhou et al. [99] reported that DSF-Cu^2+^ could up-regulate PD-L1 expression and inhibit CD8^+^ T cell infiltration and activity by suppressing poly (ADP-ribose) polymerase 1 activity and inducing glycogen synthase kinase-3β inactivation via phosphorylation at the Ser9 site. In contrast, the application of copper chelators suppressed signal transducer and activator of transcription 3 and epidermal growth factor receptor phosphorylation and enhanced the ubiquitin-mediated degradation of PD-L1 [100]. Additionally, copper chelator tetraethylenepentamine increases mouse survival by decreasing PD-L1 expression in neuroblastoma xenografts, consequently boosting the infiltration of tumor-infiltrating T cells [101]. These findings imply that copper ion can control PD-L1 expression and impact cancer immune evasion. Therefore, various approaches that decrease intratumoral copper ion levels can provide the potential to improve therapeutic efficacy in cancer immunotherapy.

#### Cancer metastasis

Cancer metastasis is a pivotal event in cancer progression, significantly contributing to cancer-related mortality. It involves the spread of cancer cells from the primary tumor to distant sites, where they establish secondary tumors, complicating treatment and reducing overall survival rates [102]. Cancer cells often acquire invasive and stem cell-like properties, utilizing biological processes such as epithelial–mesenchymal transition, essential for embryogenesis and tissue repair, to promote metastasis [103–105].

Copper ion promotes cancer metastasis through various mechanisms, including activating proliferation and metabolic enzymes [106]. The dysfunction of copper-containing secretory enzymes, including superoxide dismutase 3 (SOD3) and lysyl oxidase (LOX), profoundly impacts cancer metastasis by promoting angiogenesis, epithelial–mesenchymal transition, and cancer cell invasion [107]. Loss of SOD3 in tumor tissues increases oxidative stress, a factor linked to various aspects of cancer progression [108]. Conversely, Laukkanen [109] has shown that enhancing SOD3 activity or expression through endogenous administration of recombinant SOD3 or induction of SOD3 expression can inhibit cancer cell metastasis.

For another example, the copper-dependent amine oxidase LOX promotes cancer cell invasion and migration [110]. Extracellular copper-dependent enzymes belonging to the LOX family have been implicated in promoting a supportive extracellular matrix environment that facilitates cancer cell invasion and metastasis. The increased cross-linking of collagen fibers can enhance the stiffness of the extracellular matrix, providing physical support for cancer cells to migrate through tissues [111]. Up-regulation of LOX mRNA and protein expression has been observed in various cancer types, including head and neck squamous cell carcinoma (HNSCC) [112], breast cancer [112,113], colorectal cancer [114,115], and prostate cancer [116].

Furthermore, copper-dependent redox enzymes, such as the ErbB2-driven cell motility (MEMO1) mediator, play a crucial role in breast cancer by facilitating cell migration and invasion. MEMO1 has been demonstrated to promote cell migration by modulating cytoskeletal dynamics and facilitating the formation of adhesion sites [117,118]. Thus, copper ion is involved in crucial processes, including cellular proliferation, differentiation, angiogenesis, and cancer invasion/metastasis [119,120].

To summarize, copper ion plays a dual role in cellular processes. It necessarily serves as a catalytic cofactor for multiple physiologic processes [25]. On the other hand, excessive copper ion accumulation can lead to metabolic dysfunction and cell death [39]. This underscores the importance of maintaining a delicate balance in copper ion regulation for optimal physiological function and mitigating adverse effects. Therefore, targeting copper ion dysregulation holds promise as a strategy for cancer treatment.

## Cuproptosis is a Novel Therapeutic Target for Copper-Based Cancer Therapy

The discovery of cuproptosis represents a significant advancement in cancer treatment. While elesclomol (ES) has been reported as a drug that trigger apoptosis, its ability to induce cell death, specifically through copper ion loading, represents a novel pathway that differs from traditional types of cell death, such as apoptosis, ferroptosis, or necroptosis [121]. This process is accompanied by the activation of caspase-3 in the caspase cascade [122]. However, ES induces the accumulation of excess copper ions binding to dihydrolipoamide S-acetyltransferase (DLAT) in cancer cells results in the down-regulation of Fe-S cluster proteins and abnormal aggregation of thioctylated proteins in the TCA cycle, ultimately leading to cuproptosis of cancer cells [8]. This cascade of events provides valuable insights into the cytotoxic mechanism of copper ionophores and opens up new avenues for treating various diseases, including cancers. By targeting the specific vulnerabilities of cancer cells related to copper metabolism, novel therapeutic strategies can be developed to exploit this mechanism for more effective and targeted cancer treatments. Excess amounts of intracellular copper substances or copper ions can generate hydroxyl radicals and increase intracellular ROS levels, leading to severe oxidative damage-based apoptosis and ferroptosis [123,124]. Furthermore, copper-induced ROS and copper (II) ions can cause necroptosis through DNA damage in the cancer cells [124,125]. Copper can increase the NOD-like receptor family pyrin domain-containing protein 3, cleaved caspase-1, apoptosis-associated speck-like protein, and IL-1β protein levels, leading to cellular pyroptosis and inflammatory responses [126–128]. Therefore, copper-mediated regulated cell death (RCD) may not function as an independent mechanism of RCD and can be closely interrelated with other cell death pathways, implying that various therapeutic strategies can be developed by combining other RCD. This section mainly describes the regulatory mechanism of cuproptosis, incorporating insights from gene signaling pathways involved in the interplay between copper ions and cellular components.

First, researchers are exploring strategies to manipulate copper metabolism in cancer cells to induce cuproptosis selectively. This includes the development of copper ionophores or other biomaterials that enhance the accumulation of copper ions within cancer cells. Second, combining cuproptosis-targeting agents with other cancer therapies, such as chemotherapy or targeted therapies, may enhance therapeutic efficacy. These combination applications can provide synergistic effects and overcome resistance mechanisms in cancer cells. Finally, understanding the molecular mechanisms underlying cuproptosis in specific cancer types can enable the development of personalized treatment strategies. More effective and tailored therapies will be developed by targeting the unique susceptibilities of individual cancers related to copper ion metabolism.

### Mechanism of cuproptosis

The phenomenon of cell death is caused by excess levels of copper ion in the body called cuproptosis and is associated with changes in mitochondrial enzymes [129]. Excessive copper ion levels can cause oxidative damage to mitochondrial membranes and interfere with enzymes involved in the TCA cycle, also known as the citric acid cycle or Krebs cycle. As excess Cu^2+^ infiltrates the cell, it moves to the mitochondria and is converted to Cu^+^. These elevated Cu^+^ level disrupt both the TCA cycle and the ETC, leading to cell death due to oligomerization of lipoylated proteins and depletion of Fe-S cluster proteins [130]. A study demonstrated that cells subjected to Cu ionophores exhibited a time-dependent dysregulation of various TCA cycle-related metabolites, and the inhibition of ETC complexes I and II significantly attenuated Cu ion-induced cell death [131]. To explore mechanism of cuproptosis, researchers conducted genome-wide CRISPR-Cas9 screening. Seven genes have been discovered as regulators of cuproptosis, namely ferredoxin-1 (FDX1), lipoic acid synthetase (LIAS), lipoyltransferase 1, dihydrolipoamide dehydrogenase, DLAT, pyruvate dehydrogenase E1 subunit alpha 1, and pyruvate dehydrogenase E1 subunit beta [8] FDX1 is a pivotal enzyme with strong reducing capabilities, responsible for lipoylating 4 specific enzymes—DLAT, dihydrolipoamide succinyltransferase, glycine cleavage system protein H, and dihydrolipoamide branched chain transacylase E2 (DBT)—within the mitochondria. This indicates the critical role of lipoylation in sustaining cellular metabolic pathways and ensuring proper mitochondrial function [132].

**Fig. 1.**
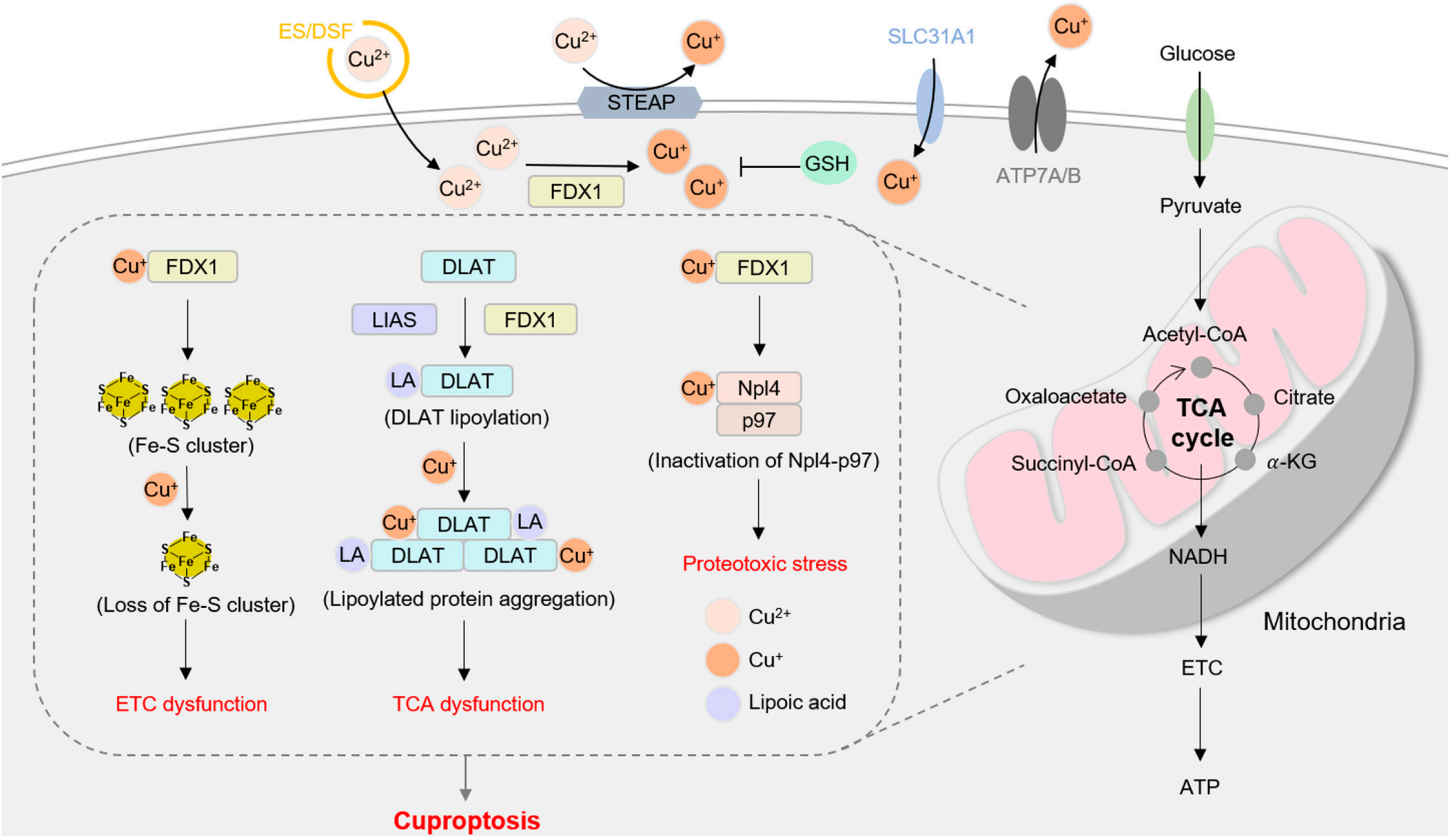
The schematic of cuproptosis mechanism. Cu ionophores like ES and DSF initially bind to extracellular copper ions, aiding in their internalization into intracellular compartments. Once inside, copper ion interacts with lipoylated mitochondrial enzymes involved in TCA cycle, such as DLAT. This interaction triggers the aggregation of these proteins. FDX1/LIAS acts as an upstream regulator of protein lipoylation, promoting the aggregation of mitochondrial proteins. This process leads to the loss of Fe-S clusters and the inactivation of Npl4-p97. These aberrant events induce proteotoxic stress, ultimately resulting in cell death. STEAP, 6-transmembrane epithelial antigen of the prostate.

Interestingly, Cu^+^ can directly bind to lipoylated TCA cycle proteins, specifically the disulfide bond on the terminal cysteine residue. This interaction triggers disulfide bond-dependent aggregation of these proteins, potentially disrupting the TCA cycle and resulting in the degradation of Fe-S cluster proteins. Also, copper ion might impede the ubiquitinated protein degradation function of valosin-containing protein (p97) by interacting with nuclear protein localization protein 4 (Npl4), which could result in its aggregation or directly binding and inhibiting its conformational transition, inducing proteotoxic stress, ultimately leading to cell death [10,133,134]. Likewise, FDX1/LIAS act as an upstream regulator of protein lipoylation, and their dysregulation facilitates the decrease of Fe-S clusters, whereas the deletion of FDX1 or LIAS results in the accumulation of α-ketoglutarate and pyruvate, leading to decrease lipoylation and cell death [135]. In addition, Tsvetkov et al. [8] investigated the impact of intracellular copper ion levels on cuproptosis by manipulating genes related to copper ion transport in vitro and in vivo. Overexpression of the copper ion importer SLC31A1 increased cell sensitivity to copper ion-induced protein aggregation and degradation of Fe-S cluster proteins. This effect was partially rescued by copper chelators and depletion of key cuproptosis regulators, FDX1 or LIAS. Consistently, these findings were validated in a mouse model of Wilson's disease mouse model with Atp7b depletion (Atp7b^−/−^). The livers of Atp7b^−/−^ mice exhibited substantial depletion of lipoylated and Fe-S cluster proteins compared to those of Atp7b^+/−^ and wild-type mice. This observation confirms that the accumulation of intracellular copper ion is associated with cuproptosis in vivo. The major processes and regulators of cuproptosis were illustrated in (Fig. 1).

### Cuproptosis-based cancer therapy

Cuproptosis, a unique cell death mechanism, has attracted significant interest in cancer research. This is because it has the potential to inhibit cancer cell proliferation and even reverse resistance to anticancer drugs, offering new treatment options. To induce cuproptosis, delivering Cu^2+^ or Cu^+^ to cancer cells is necessary. Various approaches have been developed for the intracellular delivery of copper ion, including ionophores, metal-organic frameworks (MOFs), hydrogels, and inorganic nanoparticles (Table).

**Table.** Application of cuproptosis-targeted nanomedicines used for cancer therapy

Cuproptosis-targeted nanomedicines	Nanomaterials	Cancer Type	Therapeutic effect	Combination therapy	Year	Ref.
GOx@ [Cu(tz)]	Coordination polymers	Bladder cancer	Glucose and GSH depletion enhance GOx@[Cu(tz)]-mediated cuproptosis, inducing mitochondrial protein aggregation. Elevated H_2_O_2_ levels boost type I PDT efficacy. GOx@[Cu(tz)] shows low systemic toxicity and inhibits tumor growth in mice with bladder cancers.	PDT	2022	[183]
CuET NPs	-	Non-small cell lung cancer (NSCLC)	CuET overcomes cisplatin resistance in NSCLC via cuproptosis, possessing lower reduction potential and inertness with GSH. It conquers drug resistance in A549/DDP cells, showing potent anticancer activity unaffected by GSH levels. CuET NPs induce cell death via cuproptosis and demonstrate superior biosafety in a cisplatin-resistant cancer model.	-	2022	[142]
DOX@Fe/CuTH	Hollow amorphous metal organic framework (HaMOF)	Breast cancer	DOX@Fe/CuTH demonstrates catalytic therapeutic properties triggered by the tumor microenvironment. It amplifies cellular oxidative stress by increasing H_2_O_2_ production and depleting GSH simultaneously. This induces mitochondrial dysfunction and down-regulates ATP7A expression, resulting in Cu^2+^ overload and cellular cuproptosis.	Chemotherapy	2022	[184]
Au@MSN-Cu/PEG/DSF	Mesoporous silica-coated Au nanorods	Breast cancer	Combined with PTT, Au@MSN-Cu/PEG/DSF effectively killed cancer cells, inhibited growth (inhibition rate up to 80.1%), and reduced DLAT, LIAS, and Npl4.	PTT	2023	[152]
HFn-Cu-REGO NPs	-	Glioblastoma (GBM)	HFn enables targeted delivery to GBM via TfR1. Regorafenib halts autophagy, inducing cell death. Cu^2+^ enhances drug encapsulation and triggers cuproptosis, augmenting regorafenib's efficacy against GBM.	Chemotherapy	2023	[153]
Cu_2_O@CuBTC-DSF@HA nanocomposites (CCDHs)	Metal–organic frameworks (MOFs)	Breast cancer	CCDHs degrade rapidly in acidic conditions, releasing Cu^+^ and DSF. Cu^+^ disrupts the TCA cycle, causing mitochondrial dysfunction and initiating cuproptosis. Simultaneously, Cu^+^ undergoes a Fenton-like reaction, generating ROS, and converting back to Cu^2+^. DSF chelates Cu^2+^, forming CuET, which reduces Cu^2+^ to Cu^+^ and boosts ROS production. ROS exacerbates mitochondrial damage, contributing to cuproptosis cell death and enhancing the anticancer effect synergistically.	-	2023	[185]
BSO-CAT@MOF-199 @DDM (BCMD)	MOFs	Glioblastoma (GBM)	BCMD-mediated cuproptosis can induce immunogenic cell death (ICD), promoting the infiltration of cytotoxic T lymphocytes and reversing the immunosuppressive microenvironment of glioblastoma to enhance tumoricidal immunity.	Immunotherapy	2023	[186]
Cu_2_(PO_4_)(OH) NPs	H2S-responsive copper hydroxyphosphate nanoparticles	Colon cancer	Cu_2_(PO_4_)(OH) NP, the synergy of increased endocytosis and decreased exportation results in maximum copper ion overload. Coupled with efficient copper ion release, this disrupts the mitochondrial tricarboxylic acid cycle and down-regulates iron-sulfur cluster proteins, ultimately initiating cuproptosis.	-	2023	[167]
Cu_2_O@TBP-2(PTC)	Cuprous oxide	Breast cancer	PTC triggers cuproptosis in cancer cells both in vitro and in vivo, markedly inhibits lung metastasis of breast cancer, boosts central memory T cell count in peripheral blood, and prevents tumor rechallenge.	PDT	2023	[178]
CS/MTO-Cu@AMI	MOFs	Breast cancer	Coordinated Cu^2+^ of CS/MTO-Cu@AMI triggers cuproptosis, activating AMPK-mediated PD-L1 degradation, disrupting energy supply, and amplifying oxidative stress, sensitizing chemo-immunotherapy. AMI complements by suppressing macropinocytosis and exosome release, enhancing Cu^2+^ mediated therapy.	Chemotherapy/ immunotherapy	2023	[187]
CuMoO_4_ Nanodots	-	Breast cancer	CuMoO_4_ nanodots efficiently convert light to heat, inhibiting cancer cell regulation to oxidative stress and promoting sustained photothermal synergistic ferroptosis. They trigger immune responses and induce both immunogenic cell death and cuproptosis in cancer cells.	PTT	2023	[188]
HD/BER/GOx/Cu hydrogel	Hyaluronic acid-dopamine (HD) polymer	Breast cancer	The HD/BER/GOx/Cu hydrogel reduces the frequency of dosing in local cancer therapy and mitigates invasiveness-related inconveniences. It could be used to shrink breast cancer size before surgery and suppress tumor growth in clinical settings.	CDT/CT/ST	2023	[189]
NP@ESCu	Reactive oxygen species (ROS)-sensitive polymer	Bladder cancer	Intracellular ROS-triggered release of ES and Cu synergistically eliminate cancer cells through cuproptosis and stimulate immune responses. In vitro, NP@ESCu effectively delivers Cu, inducing cuproptosis. In a mouse model of subcutaneous bladder cancer, NP@ESCu induces cuproptosis and remodels the tumor microenvironment. NP@ESCu enhances the anticancer activity of αPD-L1.	Immunotherapy	2023	[140]
TP-M-Cu-MOF/siATP7a NP	Copper-based metal organic framework (Cu-MOF)	Small cell lung cancer (SCLC)	TP-M-Cu-MOF/siATP7a demonstrated potent gene silencing, specifically hindering copper ion trafficking, enhancing copper ion intake, inducing cuproptosis, and enhancing therapeutic efficacy in mice with SCLC brain metastasis tumors.	Gene therapy	2023	[190]
Au25(NAMB)18 NCs-Cu^2 +^ @SA/NHGs	Nanohybrid gels	Hepatocellular carcinoma (HCC)	The release of Cu^2+^ from the nanohybrid gels induced cuproptosis and catalyzed H_2_O_2_ to generate O_2_, enhancing PDT. Cu^2+^ depleted GSH, forming Cu^+^, which produced hydroxyl radicals to kill cancer cells, synergizing with enhanced PDT and CDT.	PTT/PDT/CDT	2023	[191]
CCNAs	Copper-coordinated nanoassemblies	Prostate cancer	The inclusion of Cu^2+^ in the CCNAs not only amplified the photodynamic process by catalyzing oxygen generation but also facilitated the aggregation of toxic mitochondrial proteins, ultimately triggering cuproptosis in cancer cells.	PDT/ immunotherapy	2024	[192]
CuSiO_3_@Au-Pd NMs	Multifunctional copper phyllosilicate-based nanomotors	Breast cancer	The generation of ·OH by CuSiO_3_@Au-Pd NMs through Fenton-like reactions and the thermal energy produced by the photothermal effect cooperatively induced apoptosis in cancer cells. Moreover, these thermal gradients facilitated the movement of CuSiO_3_@Au-Pd NMs via thermophoresis, promoting deeper penetration into tumor tissue.	PTT/CDT	2024	[193]
CuO_2_/DDP@SiO_2_	Silica-coated copper peroxide (CuO_2_)	Hepatocellular carcinoma (HCC)	Depleting GSH sensitizes cancer cells to CuO_2_/DDP@SiO_2_-induced cuproptosis, causing lipoylated mitochondrial protein aggregation. In vitro, reduced GSH binding boosts intracellular cisplatin levels. CuO_2_ down-regulates MRP2 via O_2_-dependent HIF-1 inactivation, blocking cisplatin efflux and enhancing its anticancer effect both in vitro and in vivo.	Chemotherapy/CDT	2024	[194]
HA-CuH-PVP (HCP).	HA combined with PVP	Breast cancer	HCP effectively damages tumor cells by generating Cu^+^ and hydrogen (H_2_), inducing apoptosis and cuproptosis, and shows promising in vivo antitumor and metastasis-inhibiting potential.	Apoptosis	2024	[179]
Cu(I) NP	Cu(I) self-assemble with a ROS-sensitive polymer	Pancreatic cancer	ROS-sensitive copper complex nanoparticles (Cu(I) NP) that deliver copper complexes to cancer cells, inducing cuproptosis. In a pancreatic cancer mouse model, Cu(I) NP accumulated in tumors, inhibited growth, and enhanced immune responses.	Immunotherapy	2024	[180]
CuP/Er	Nanoscale coordination polymers (NCPs)	Breast cancer/ colon cancer	CuP/Er nanoparticles codeliver copper and erastin to synergize cuproptosis and ferroptosis, enhancing T cell response and effectively regressing cancers with immune checkpoint blockade.	Ferroptosis/ immunotherapy	2024	[181]
MetaCell	Thermosensitive liposomal bimetallic Fe-Cu MOFs (Lip@Fe-Cu-MOFs)	Breast cancer	MetaCell system integrates cuproptosis and ferroptosis using Fe-Cu-MOFs to catalyze Fenton-like reactions, producing radicals and depleting GSH in tumor cells, effectively eliminating solid tumors, preventing recurrence, and extending survival.	PTT/ferroptosis	2024	[182]

Among them, ionophores can bind metal ions reversibly under physiological conditions. This not only promotes the transmembrane transport of metal ions in the electrically neutral and lipophilic state but can also change the biodistribution of metal ions by releasing them under specific stimuli, such as local low metal ion concentrations or biological reduction [130,136,137]. ES, DSF, and NSC319726 are all considered copper ionophores with the potential to kill cancer cells [138,139]. Specifically, NP@ESCu, a nanoparticle utilizing the traditional ionophore ES, has facilitated the targeted delivery of copper ion to mitochondria [140]. ES can reversibly bind with Cu^2+^, resulting in the formation of an ES–Cu^2+^ complex. This complex facilitates the direct transportation of copper ion into mitochondria. Within the mitochondria, FDX1 is responsible for reducing Cu^2+^ to Cu^+^ and subsequently releasing it. The increased level of Cu^+^ directly binds to lipoylated DLAT, leading to the lipoylated proteins aggregation and loss of Fe-S cluster proteins, ultimately resulting in cuproptosis [8,130,137].

Additionally, DSF, which has antiproliferative effects, has shown promise for cancer treatment by inducing cuproptosis beyond its role as a copper ion carrier [8,141]. DSF has also been studied for its ability to reverse drug resistance, making cancer cells more sensitive to chemotherapy [142]. Furthermore, DSF has been found to have immunomodulatory effects, potentially enhancing the body’s immune response against cancer cells [143].

Despite promising preclinical findings demonstrating beneficial anticancer effects through copper chelation or induction of copper ion-dependent cell death via copper ionophores, several limitations remain in the clinical translation, including their lack of specificity and short half-life. Developing novel approaches is essential to address these limitations and advance the clinical translation of treatments targeting copper-dependent mechanisms for effective cancer therapy.

## Cuproptosis-Targeted Nanomedicines for Combination Cancer Therapy

For effective cancer therapy, cuproptosis-based cancer treatment still needs to overcome several limitations, including the selective increase of copper ion levels in cancer cells, preventing copper ion damage to healthy cells, and extending the duration of cuproptosis [18,19]. Nanomedicine emerges as a crucial solution for tackling this challenge, given its effectiveness in enhancing drug solubility, prolonging circulation time, enabling targeted drug delivery, and minimizing unexpected side effects [144–146]. Consequently, the recent elucidation of this phenomenon has led to significant progress in developing nanomaterials based on cuproptosis and has shown promising progress in applications for cancer eradication. For example, copper and copper oxide nanoparticles have garnered significant attention in cancer biology, presenting a wide array of advantages such as enhancing drug stability, ensuring appropriate biodistribution, refining therapeutic index, and facilitating targeted delivery of active agents to specific sites through active or passive targeting mechanisms [147–151].

In another study, a photothermal-activated nanosystem infused with copper (Au@mesoporous silica nanoparticles [MSN]-Cu/polyethylene glycol [PEG]/DSF) has been engineered, comprising mesoporous silica-coated gold nanorods (Au@MSN) functioning as photothermal agents [152]. This system exhibits excellent loading capacity for Cu^2+^ and the anticancer drug DSF, facilitating synergistic treatment involving cuproptosis, apoptosis, and photothermal therapy (PTT). In the following section, we provide the recent strategies employing anticancer nanomaterials based on cuproptosis and categorize potential synergistic treatment approaches for improved cancer treatment.

Since the concept of cuproptosis, increasing evidence has demonstrated the anticancer potential of cuproptosis, and much effort has been devoted to the design and development of various copper-based nanomaterials for cancer treatment. The nanomaterials up-regulate local copper ion concentration by delivering copper ions to the tumor site. Increasing copper ion levels represents a novel approach for copper-ion-interference therapy, disrupting copper homeostasis and addressing diseases through various cell death pathways. For example, Jia et al. [153] developed a brain-targeted nanoplatform (HFn-Cu-REGO NPs), incorporating human H-ferritin (HFn), regorafenib, and Cu^2+^, enabling site-specific delivery and modulation of autophagy and cuproptosis against glioblastoma multiforme (GBM) (Fig. S3A). Effective management of persistent GBM often requires prolonged chemotherapy postsurgery to eliminate residual cancerous tissues [154]. While temozolomide is the primary chemotherapeutic agent for GBM therapy, its treatment outcomes remain unsatisfactory [155].

However, regorafenib, an oral multikinase inhibitor, exhibits significantly superior effects in suppressing GBM compared to temozolomide [156]. HFn, due to its selective accumulation in GBM facilitated by transferrin receptor 1 (TfR1)-mediated active targeting and pH-responsive delivery characteristics, serves as an ideal carrier for targeted drug delivery [157]. Regorafenib inhibits autophagosome-lysosome fusion, leading to lethal autophagy arrest in GBM cells [156]. Cu^2+^ facilitates the encapsulation of regorafenib within HFn through coordination interaction and disrupts copper homeostasis, inducing cuproptosis (Fig. S3B) [8,158]. Dynamic light scattering analysis confirmed the narrow size distribution of HFn with a mean diameter of approximately 15.5 nm (Fig. S3C).

To explore the potential involvement of cuproptosis in HFn-Cu-REGO-mediated cell death, immunofluorescence analysis was conducted to assess the endogenous levels of lipoic acid and FDX1 in GBM cells treated with regorafenib, HFn-Cu, and HFn-Cu-REGO NPs for 24 h. The immunofluorescence results revealed a notable accumulation of FDX1 in the groups treated with HFn-Cu and HFn-Cu-REGO NPs (Fig. S3D). Treatments involving HFn-Cu and HFn-Cu-REGO NPs demonstrated a significant rise in intracellular copper ion levels within GBM cells (Fig. S3E and F). Moreover, these treatments induced fluctuations in the expression levels of various Cu^2+^ transporters in both U251 and U87 cells (Fig. S3G and H). As shown in (Fig. S3I), the group treated with HFn-Cu-REGO NPs exhibited significant cancer damage, attributed to efficient delivery and therapeutic synergy between regorafenib and Cu^2+^. Additionally, treatment with HFn-Cu and HFn-Cu-REGO NPs intensified staining of lipoic acid and FDX1 in brain tumor tissues (Fig. S3J). Consequently, mice treated with HFn-Cu-REGO NPs exhibited delayed tumor growth and the lowest bioluminescence levels compared to other treatment groups (Fig. S3K). This synergy with regorafenib-mediated lethal autophagy arrest enhances the therapeutic impact against GBM [159].

In other cases, Zhao et al*.* prepared hydrogen sulfide (H_2_S)-responsive copper hydroxyphosphate nanoparticles (Cu_2_(PO_4_)(OH) NPs) to enhance cellular uptake and reduce the efflux of copper ions [160,161]. This approach can achieve copper ion overload because excess intracellular copper ions tend to be pumped out of the cells by the copper exporter ATP7A [162,163]. Upon exposure to an H_2_S-rich microenvironment in colon cancer, Cu_2_(PO_4_)(OH) NPs transform smaller copper sulfide NPs, enhancing cellular uptake [164–166]. This process improves Fenton activity and copper ion dissociation [167]. ROS generated through the Fenton reaction activate inflammasomes and Caspase-1 proteins, leading to gasdermin D cleavage and induction of pyroptosis [168–171]. These ROS also affect mitochondrial function and down-regulate the copper exporter ATP7A, further reducing copper excretion. The synergy of enhanced endocytosis and reduced exportation results in maximal copper ion overload. This, coupled with the efficient release of copper ions, disrupts the mitochondrial TCA cycle and down-regulates Fe-S cluster proteins, ultimately inducing cuproptosis. Since both pyroptosis and cuproptosis are effective mechanisms for cell death induction, this research offers a novel approach for achieving effective cancer-targeted therapy through H_2_S-activated copper ion overload using simple Cu_2_(PO_4_)(OH) NPs [167]. The findings demonstrated the potential of inducing cuproptosis in cancer cells using copper-based nanomaterials as a promising strategy for cancer treatment.

Because cancer cells are commonly heterogeneous, relying solely on chemotherapy may prove less effective and less tolerable for certain cancers. Thus, implementing combination therapies through various methods is essential to enhance treatment outcomes [172,173]. Specifically, the discovery of cuproptosis contributing to tumor immunogenicity suggests its potential to augment the effectiveness of immune checkpoint blockade (ICB) therapy [174,175]. ICB has emerged as a pivotal approach in cancer immunotherapy, enhancing and activating the anticancer immune responses in the body [176]. Recent studies indicated that reduced intracellular copper ion levels promoted ubiquitin-mediated degradation of PD-L1 in cancer cells, increasing antitumor immunity [15]. Consequently, there is the possibility of a synergistic effect between cuproptosis and ICB therapy.

**Fig. 2.**
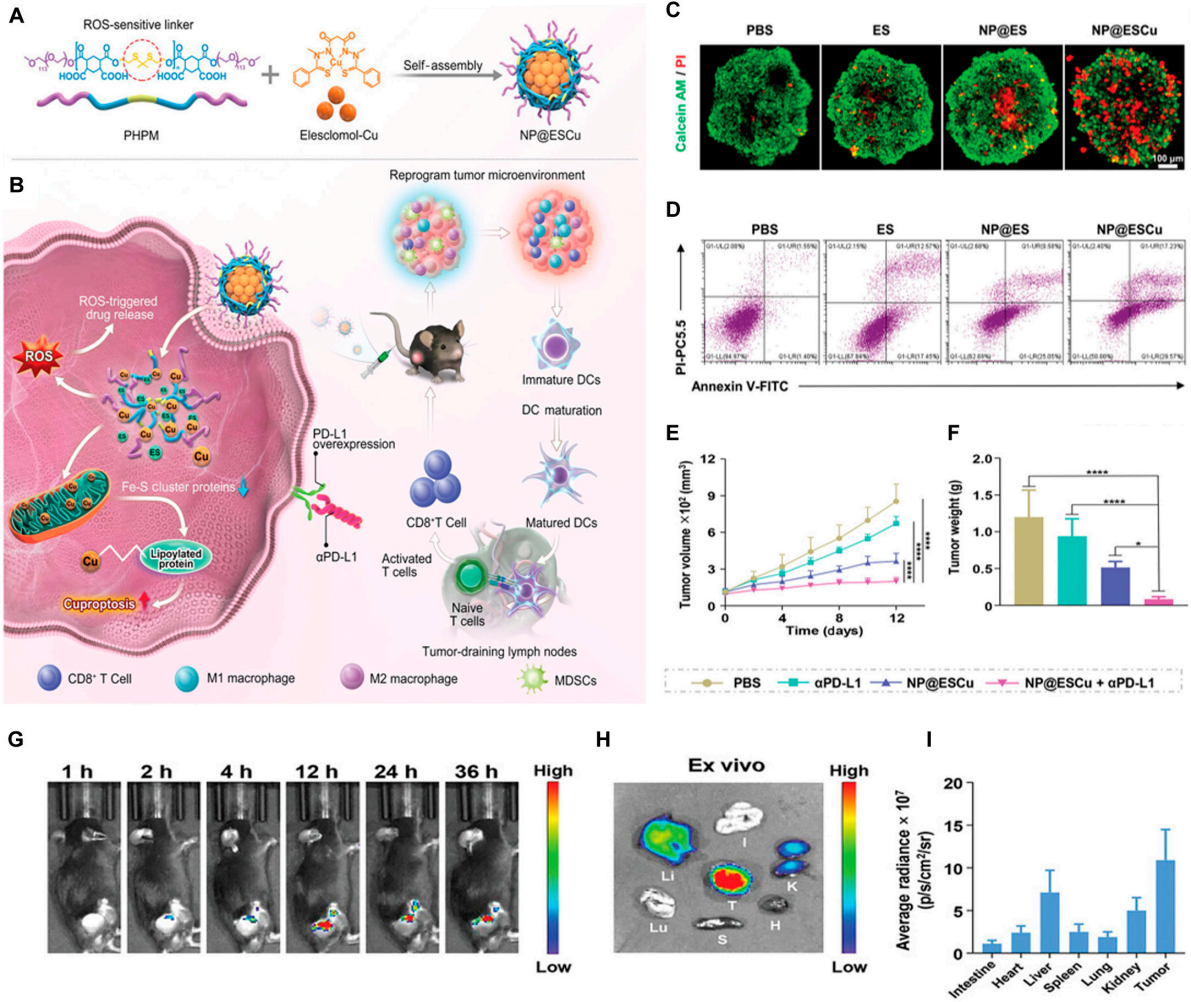
Cuproptosis induction via ROS-responsive nanoparticles incorporating ES and copper ion in conjunction with αPD-L1 for augmented cancer immunotherapy. (A) Schematic illustration of NP@ESCu to induce cuproptosis. (B) Design of converting immune “cold tumors” to “hot tumors” through NP@ESCu induces cuproptosis, eliciting strong antitumor immune responses in vivo, further maximized by combining with αPD-L1. (C) Representative CLSM images of 3D cell spheroids from BIU-87 cells subjected to various treatments, stained with calcein-AM/PI. Scale bars: 100 μm. (D) Representative flow cytometry profiles illustrating the apoptotic rate of BIU-87 cells under various treatment treatments. (E) Tumor growth curves and (F) Ex vivo tumor weight of mice under various treatments. (G) Biodistribution following intravenous injection of NP@ESCu@Cy7.5 at different time points. (H) Ex vivo imaging and (I) Mean fluorescence intensity of NP@ESCu@Cy7.5 in major organs and tumor (I, intestine; S, spleen; H, heart; Lu, lung; K, kidney; Li, liver; T, tumor). Reproduced from [140] with permission from Wiley-VCH, Copyright 2023.

Guo et al. [140] designed a ROS-sensitive polymer known as PHPM has been developed for the coencapsulation of ES and copper ion, resulting in the formation of nanoparticles termed NP@ESCu. The design involves an amphiphilic biodegradable polymer termed PHPM, as shown in (Fig. 2A). PHPM is notable for containing ROS-sensitive thioketal bonds in its main chain, along with pendant pairs of carboxylic acids. ES and Cu^+^ are promptly released upon entering cancer cells, triggered by the abundance of intracellular ROS. Increasing the presence of copper ionophores like ES in tumor tissues can expedite the delivery of copper ion into cancer cells, enhancing the antitumor effect through cuproptosis [177].

Additionally, transporting copper ion into cancer cells with ionophores can elevate PD-L1 expression (Fig. 2B) [15]. The anticancer activity of NP@ESCu was evaluated using a live–dead staining assay on 3-dimensional (3D) tumor spheroids, demonstrating that the highest number of dead cells (indicated by red staining) was observed in the 3D tumor spheroids treated with NP@ESCu (Fig. 2C). Furthermore, the apoptotic rate of BIU-87 cells treated with various drugs was investigated using an Annexin V-FITC and propidium iodide (PI) double-staining assay. The findings revealed that the apoptotic rate of BIU-87 cells treated with NP@ESCu was 44.9%, surpassing that of cells treated with ES (29.8%) by 1.5 times and exceeding that of cells treated with NP@ES (35.5%) by approximately 1.3 times in a culture medium containing CuCl_2_ (0.1 μm) (Fig. 2D). The anticancer effect of ES was minimal on day 12, similar to NP@ES in vivo. In contrast, NP@ESCu exhibited the most significant anticancer effect (Fig. 2E and F). Remarkably, even 36 h postinjection of NP@ESCu@Cy7.5, a robust fluorescence signal persisted at the tumor site, suggesting rapid accumulation and prolonged retention of NP@ESCu@Cy7.5 in the tumor microenvironment (Fig. 2G). After 36 h, mice were sacrificed, and NP@ESCu@Cy7.5 biodistribution was assessed ex vivo. The strongest fluorescence signal was observed in tumors, indicating effective tumor targeting (Fig. 2H and I). Taken together, NP@ESCu demonstrated rapid and efficient targeting and accumulation at tumor sites, highlighting its potential for clinical applications. Hence, combining copper ionophores with immunotherapeutic agents like anti-PD-L1 antibody (αPD-L1) may offer a more effective cancer therapy strategy.

**Fig. 3.**
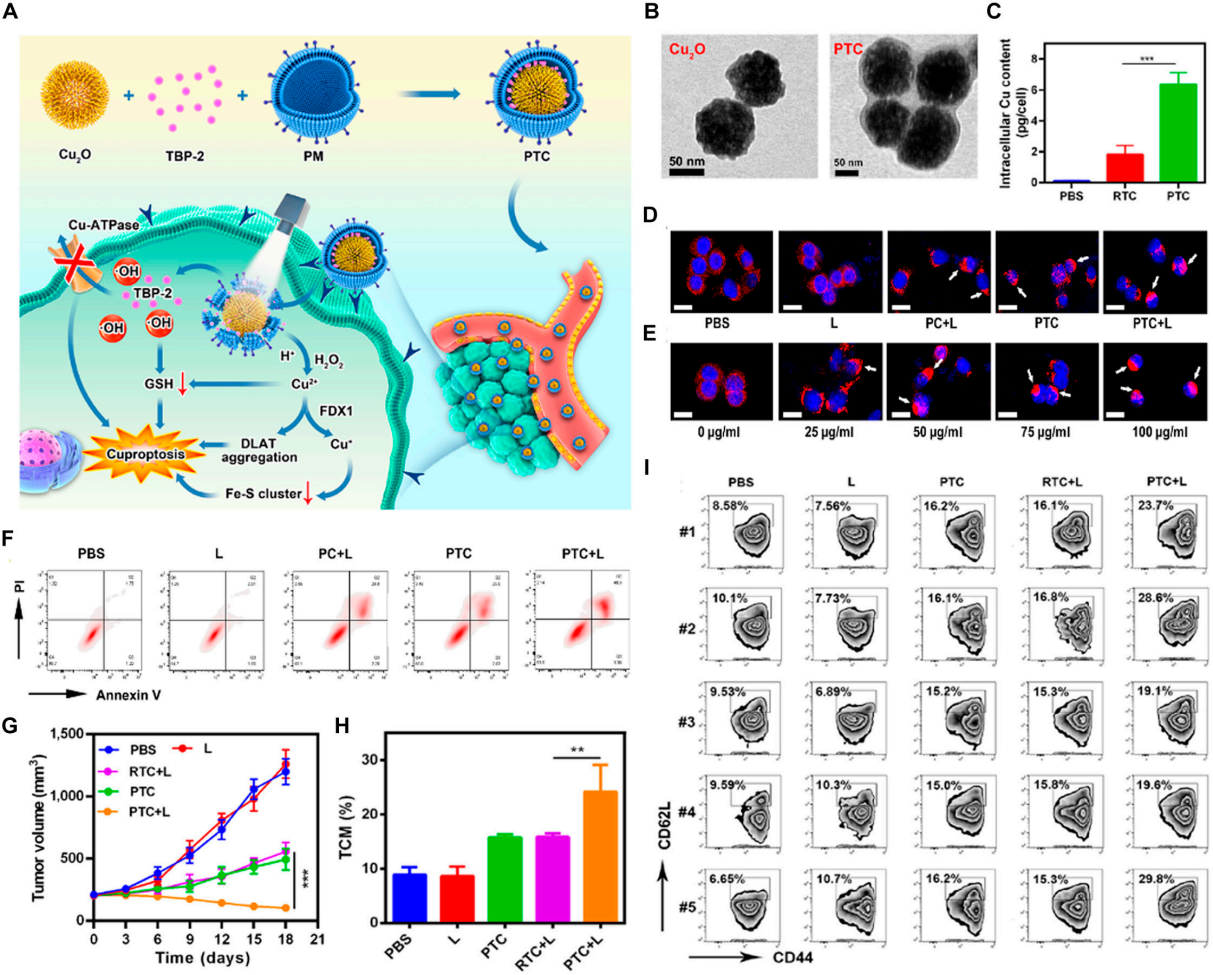
Enhancing cuproptosis in breast cancer metastasis inhibition and rechallenge resistance through combination therapy with type-I AIE photosensitizer loaded biomimetic system. (A) Schematic illustration of a type-I AIE photosensitizer integrated into a biomimetic system to trigger cancer cuproptosis. (B) Transmission electron microscopy images of Cu_2_O and PTC. Scale bars: 50 nm. (C) Alterations in the concentration of copper ions within cells following incubation with various formulations. (D) DLAT fluorescence images of cancer cells posttreatment as specified. DLAT aggregation is highlighted by white arrows. PC: PM-coated Cu_2_O without TBP-2. Scale bars: 15 μm. (E) DLAT fluorescence images of cancer cells post PTC+L treatment with varying concentrations of Cu_2_O. DLAT aggregation is highlighted by white arrows. Scale bars: 15 μm. (F) Analysis of cellular apoptosis in 4T1 cells following treatment with various formulations. (G) Changes of tumor volume in mice after undergoing diverse treatments for tumor metastasis. (H and I) The ratio of central memory T cells (TCM, CD62L^+^, CD44^+^) to CD3^+^, CD8^+^ T cells in the bloodstream 14 d post PTC treatment for tumor rechallenge assessed via flow cytometry. Reproduced from [178] with permission from American Chemical Society, Copyright 2023.

Ning et al*.* [178] developed a cuprous oxide nanoparticle (Cu_2_O)/TBP-2 cuproptosis sensitization system coated with platelet vesicles, abbreviated as PTC. PTC was formed through the physical extrusion of the AIE photosensitizer (TBP-2), Cu_2_O, and platelet vesicle. PTC exhibits enhanced long-term blood circulation and tumor-targeting capabilities (Fig. 3A). As shown in (Fig. 3B), the PTC surface features a detailed gray cell membrane structure, indicating successful encapsulation with a platelet membrane. Also, the copper ion content within tumor cells notably surged in the PTC group within 2 h, reaching around 6 pg/cell. In contrast, the intracellular copper ion content in tumor cells of the erythrocyte membrane-coated RTC group remained approximately at 2 pg/cell. This observation further validated the tumor-targeting efficacy of PTC (Fig. 3C). Then, the fluorescence of the dihydrolipoyllysine-residue acetyltransferase component of the pyruvate dehydrogenase complex, mitochondrial DLAT, and the expression of cuproptosis-related proteins were examined in tumor cells treated with different materials. During cuproptosis, copper ions directly bind to lipoylated components of the TCA cycle, causing aggregation of the fatty acylated proteins and loss of Fe-S proteins. Both the PC+L and PTC groups exhibited varying degrees of DLAT aggregation, with the PTC+L group showing higher DLAT aggregation. Additionally, an increase in PTC concentration correlated with increased aggregation (Fig. 3D and E). PTC+L treatment is also capable of inducing apoptosis in tumor cells (Fig. 3F). Subsequently, they assessed the antitumor efficacy of PTC in vivo, observing a partial tumor suppression rate in both the PC+L and PTC groups. Notably, the PTC+L group demonstrated pronounced tumor growth when exposed to light, indicating that PTC exhibited a high tumor growth under light conditions (Fig. 3G). In addition, 14 d after the initial treatment, peripheral blood was collected from mice, and the ratio of central memory T cells (TCM, CD3^+^, CD8^+^, CD62L^+^, and CD44^+^) to CD3^+^, CD8^+^ T cells was analyzed using flow cytometry. The findings revealed that the TCM content was most elevated following PTC+L treatment, suggesting a robust immune memory response triggered by PTC+L therapy (Fig. 3H and I). Thus, PTC treatment effectively targets and induces cuproptosis in cancer cells both in vitro and in vivo, significantly suppressing lung metastasis of breast cancer and increasing central memory T cell counts in peripheral blood, resulting in preventing recurrence after tumor rechallenge. This strategic design of combination therapy induces various forms of cell death linked to ROS and offers significant benefits for cancer treatment.

Although cuproptosis has received enormous attention for cancer treatment, specific activation of cuproptosis with copper utilization in the tumor microenvironment remains challenging. To address this limitation, He et al. [179] developed HA-coated acid-degradable copper hydride (HCP) nanoparticles using microfluidic synthesis. Surface HA on HCP enabled targeting CD44 receptors on 4T1 breast tumor cells, resulting in internalized via receptor-mediated endocytosis and inducing significant tumor cell damage by simultaneously releasing Cu^+^ ions and hydrogen (H_2_). The Cu^+^ ions triggered apoptosis by Fenton-like reactions and induced cuproptosis by aggregating mitochondrial proteins. Additionally, released H_2_ increased both cell death by promoting mitochondrial dysfunction and intracellular redox imbalance. Finally, HCP significantly inhibited tumor growth and prevented lung metastases, showing potent therapeutic efficacy by copper-based antitumor materials.

**Fig. 4.**
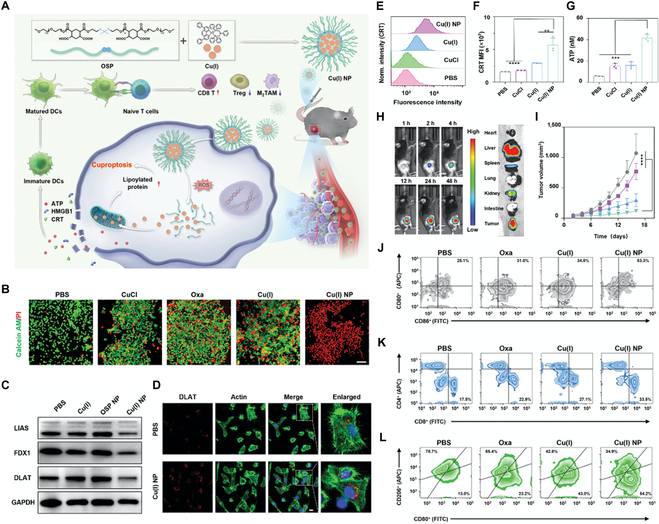
Stimulus-responsive copper complex nanoparticles as inducers of cuproptosis for enhanced cancer immunotherapy. (A) Schematic illustration of Cu(I) NP preparation and mechanism for cuproptosis-induced immunotherapy. (B) CLSM images of live/dead staining in MIAPACA-2 cells following different treatments. Scale bars: 50 μm. (C) Western blot analysis of LIAS, FDX1, and DLAT expression levels in MIAPACA-2 cells. (D) CLSM images showing DLAT aggregation in MIAPACA-2 cells following Cu(I) NP treatment. Scale bars: 20 μm. (E) Flow cytometry analysis of CRT exposure under different treatments and (F) corresponding quantification. (G) Quantitative analysis of ATP levels in the supernatant of MIAPACA-2 cells under various treatments. (H) In vivo biodistribution of Cy7.5@Cu(I) NP by IVIS and ex vivo imaging of tumors and major organs 48 h postadministration. (I) Tumor growth inhibition curve in mice following various treatments. (J) Flow cytometry results of CD80^+^ CD86^+^ dendritic cells gated on CD11c^+^ in tumors. (K) Flow cytometry results of CD8^+^ and CD4^+^ T cells gated on CD3^+^ cells in tumors. (L) Flow cytometry profiles of M1 and M2 macrophage phenotypes at tumor sites under various treatments. Reproduced from [180] with permission from Wiley-VCH, Copyright 2024.

Hu et al. [180] developed stimuli-responsive copper complex nanoparticles (Cu(I) NP) designed to deliver copper (I) ions into cancer cells for cuproptosis-based cancer immunotherapy (Fig. 4A). Dinitrogen-diphosphine chelated Cu(I) complex ([Cu(2,9-dimethyl-1,10-phenanthroline)(N,N-bis((diphenylphosphaneyl)methyl)pyridin-3-amine)](BF_4_)) was synthesized and then nanoparticulated with a ROS-sensitive polymer through electrostatic and hydrophilic interactions, resulting in forming Cu(I) NP. Thioketal moieties in ROS-sensitive polymer enabled rapid dissociation of Cu(I) NP after incubation with H_2_O_2_, resulting in 91.4% of Cu(I) being released within 36 h. Cu(I) NP showed time-dependent cellular uptaken in human pancreatic cancer cells, MIAPACA-2, and effective penetration in human pancreatic cancer cells (PANC-1)-based 3D multicellular tumor spheroids. Cu(I) NP exhibited a higher antitumor effect in different pancreatic cancer cell lines, wherein the half-maximal inhibitory concentration (IC_50_) value of Cu(I) NP was 0.16 μM only, as showed 1/33 of oxaliplatin (Oxa). Furthermore, live–dead cell staining on MIAPACA-2 cells showed that Cu(I) NP had a superior cell-killing effect compared to CuCl, Oxa, or Cu(I) (Fig. 4B). Notably, Cu(I) NP-treated MIAPACA-2 cells showed significantly reduced FDX1, LIAS, and DLAT protein expression level, supporting an induction of cuproptosis (Fig. 4C). Additionally, confocal laser scanning microscopy (CLSM) revealed pronounced DLAT aggregation in Cu(I) NP-treated cells compared to phosphate-buffered saline (PBS) controls (Fig. 4D). Next, they demonstrated that Cu(I) NP could induce immunogenic cell death (ICD). Cu(I) NP-treated cells showed 2.4- and 2.8-fold higher CRT translocation than those treated with CuCl and PBS, respectively (Fig. 4E and F). Adenosine triphosphate (ATP) release analysis showed that Cu(I) NP-treated cells released 41.4 nM of ATP, significantly higher than that of CuCl (15.3 nM) and PBS (5.9 nM) (Fig. 4G). In a subcutaneous PANC-02 model mice model, Cy7.5-labeled Cu(I) NP (Cy7.5@Cu(I) NP) was mainly accumulated in the liver and tumor tissue after intravenous injection (Fig. 4H). Furthermore, Cu(I) NP exhibited effectively reduced tumor growth compared to other treatments (Fig. 4I). Cu(I) NP-treated mice showed an increased proportion of mature dendritic cells (CD80^+^ CD86^+^) in tumor and spleen tissues, indicating enhanced antigen presentation and dendritic cell maturation (Fig. 4J). Furthermore, CD8^+^ T cell infiltration was 1.5 times higher in Cu(I) NP-treated tumors than those treated with Oxa, showing enhanced antitumor immunity (Fig. 4K). Finally, Cu(I) NP elicited higher transformation of the M2 phenotype of macrophages into the M1 phenotype in tumor tissue, indicating reprogramming of the immunosuppressive tumor microenvironment (Fig. 4L). These studies showed that specific activation of copper-based nanomedicines in tumor microenvironment could enhance anticancer effects by cuproptosis.

**Fig. 5.**
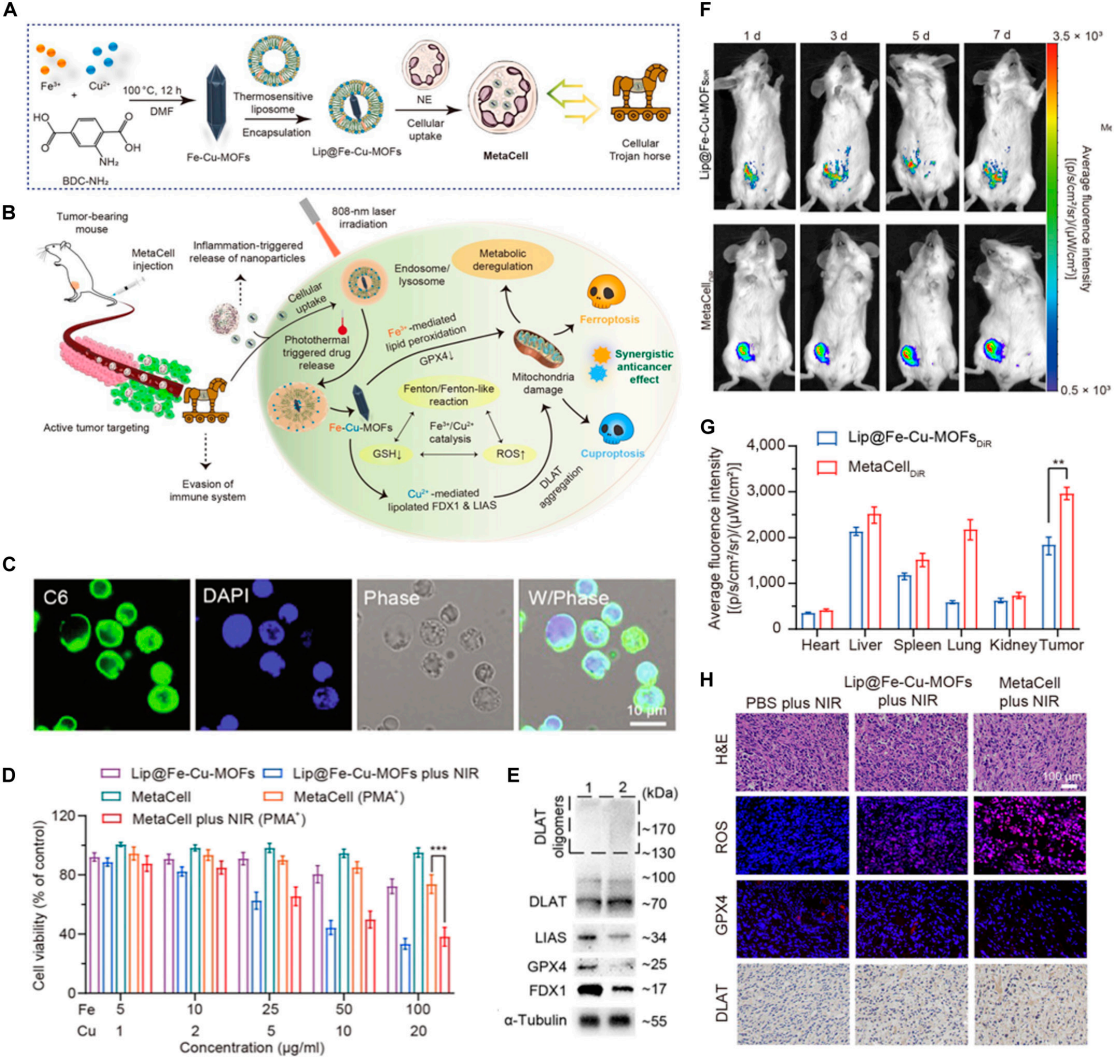
Lip@Fe-Cu-MOFs-internalized neutrophil (MetaCell)-mediated synergistic cuproptosis and ferroptosis-based treatment of malignancies. (A) Design and preparation of MetaCell. (B) Schematic illustration of synergistic anticancer effects of cuproptosis and ferroptosis induced by MetaCell. (C) CLSM images of MetaCell after incubation with Lip@Fe-Cu-MOFs. Scale bars: 10 μm. (D) In vitro anticancer effects of MetaCell on 4T1 cells under various treatments. (E) Western blot analysis of DLAT, LIAS, FDX1, and GPX4 expression levels in 4T1 cells, wherein (1) was treated with neutrophils or (2) MetaCell with phorbol-12-myristate-13-acetate (PMA) and NIR irradiation. (F) In vivo tumor targeting ability of MetaCell targeting in 4T1 tumor-bearing. (G) Biodistribution of DiR-labeled Lip@Fe-Cu-MOFs and MetaCell in tumors and major organs on 7 d postadministration. (H) Representative tumor tissue section images stained with hematoxylin and eosin, ROS, GPX4, and DLAT after treatment. Scale bars: 100 μm. Reproduced from [182] with permission from the American Association for the Advancement of Science, Copyright 2024.

Li et al. [181] developed a bifunctional CuP/Er nanoparticle that combines copper ions and peroxide in the core with erastin (Er) on the shell, designed to synergize the effects of cuproptosis and ferroptosis for enhanced cancer treatment. The CuP/Er nanoparticles sensitized 4T1 breast tumor cells to cuproptosis by disrupting their reliance on aerobic glycolysis and inhibiting the TCA cycle, leading to the oligomerization of lipoylated TCA proteins in mitochondria. Simultaneously, CuP/Er promoted ferroptosis by increasing ROS production and creating an intracellular redox imbalance. This dual action depleted glutathione (GSH), enhanced lipid peroxidation, and caused severe mitochondrial damage, resulting in significant tumor growth inhibition in mouse models of 4T1 breast and MC38 colon cancer. Interestingly, CuP/Er induced immunogenic cell death, improving antigen presentation and up-regulating PD-L1 expression in tumor cells. The combination of CuP/Er with a αPD-L1 showed potent tumor regression and prevented metastasis by synergizing T cell proliferation and reinvigoration. As another combination approach of cuproptosis and ferroptosis, Chen et al. developed a thermosensitive liposomal bimetallic Fe-Cu MOFs (Lip@Fe-Cu-MOFs, named MetaCell) to simultaneously activate cuproptosis and ferroptosis by laser irradiation [182]. Fe-Cu-MOFs were first synthesized using iron and copper metal ions via coordination bonding with organic ligands. Then, Fe-Cu-MOFs were encapsulated into thermosensitive liposomes, followed by delivering them into live neutrophils, resulting in MetaCell (Fig. 5A). MetaCell could infiltrate the tumors as well as respond to inflammation, releasing Fe-Cu-MOFs and inducing photo-triggered cuproptosis with ferroptosis (Fig. 5B). The C6-labeled Lip@Fe-Cu-MOFs showed intracellular localization in MetaCell that they were rapidly uptaken by neutrophils at 2 h posttreatment and distributed throughout the cytoplasm (Fig. 5C). MetaCell combined with phorbol-12-myristate-13-acetate, which a compound for inducing oxidative stress and inflammation, demonstrated a strong anticancer effect under near-infrared (NIR) irradiation (Fig. 5D). Moreover, MetaCell led disruption of intracellular metal homeostasis after laser irradiation, decreasing in FDX1 and LIAS levels and inducing cell death by cuproptosis (Fig. 5E). After intravenous injection into 4T1 orthotopic breast tumor model, DiR-labeled MetaCell showed superior tumor-targeting ability compared to DiR-labeled Lip@Fe-Cu-MOFs for 7 d, resulting in exhibiting 1.55-fold higher fluorescence intensity than DiR-labeled Lip@Fe-Cu-MOFs (Fig. 5F and G). Consequently, MetaCell (2 × 10^7^ cells per mouse) showed effective tumor ablation effects, after exposure to NIR laser irradiation (808 nm, 1.5 W/cm^2^) for 5 min, 24 h postinjection. Notably, MetaCell showed higher antitumor effects compared to Lip@Fe-Cu-MOFs, indicating enhanced antitumor effects by neutrophil. Furthermore, DLAT levels increased while GPX4 levels decreased, indicating that the treatment exerted its antitumor effect through a synergistic cuproptosis-ferroptosis mechanism by Lip@Fe-Cu-MOFs (Fig. 5H).

Although various combination strategies of cuproptosis-targeted nanomedicines showed enhanced therapeutic efficacy in various cancer treatments, these studies collectively suggest further researches are needed in relation to the combination of different regimes and cell death mechanisms with copper strategies for further optimizing therapeutic efficacy.

## Conclusions and Future Perspectives

Cuproptosis is one of the programmed cell deaths triggered by the accumulation of copper ions in the cells. This concept has gained attention in cancer therapy as a potential strategy for enhancing treatment efficacy. Since cuproptosis was introduced in 2022, numerous research studies have reported its cellular regulatory mechanisms and signaling pathways. Furthermore, numerous studies have demonstrated the close relationship between cancer and copper, as disrupted copper homeostasis is frequently observed in various malignant cancers, wherein dysregulated copper homeostasis can drive cancer metastasis, immune evasion, angiogenesis, and drug resistance. Thus, harnessing cuproptosis may provide novel strategies for cancer treatment to overcome resistance by apoptosis and avoid the risk of necrosis-related inflammation. This review comprehensively introduces dysregulated copper homeostasis in cancer and cuproptosis-mediated therapeutic strategies in nanomedicines to provide insights into novel designs and therapeutic applications.

Despite the therapeutic potential of cuproptosis-based nanomedicines for cancer treatment, several limitations remain to be addressed through in-depth studies. Firstly, the mechanism of cuproptosis is still being explored, requiring the exact mechanism with associated molecular pathways. Moreover, limited specific biomarkers in pathological conditions that can evaluate therapeutic efficacy in cancer treatment may hinder applications of cuproptosis-based nanomedicines in the clinic. The complicated and compensated cuproptosis mechanism can provide novel designs of nanomedicines and therapeutic strategies in combination with other regimes. Secondly, establishing the effective range of intracellular copper ion concentration for cuproptosis may closely relate to eliciting therapeutic efficacy and reducing adverse side effects. Because cuproptosis based on intracellular copper metabolism, it can induce cytotoxicity in both cancer cells and normal cells, resulting in acting as a double-edged sword. Thus, control of intracellular copper ion concentration should be carefully optimized. Thirdly, nanomedicines designed for copper ion delivery to cancer cells are carefully considered for inducing the physicochemical properties of copper. For example, copper/copper oxide nanoparticles could provide copper ions in the cells, but copper ions induced a Fenton reaction using hydrogen peroxide, generating a cytotoxic hydroxyl radical. Furthermore, the intracellular turnover mechanism of Cu^2+^ to Cu^+^ in target cancer cells should be considered for an effective cuproptosis based on DLAT aggregation and Fe-S cluster loss caused by excessive Cu^+^ in cancer cells. Thus, a rational design and related dose optimization are needed for nanomedicines to induce an effective cuproptosis. Lastly, various combination approaches using other therapeutic regimes based on different cell death mechanisms can be considered to improve the anticancer efficacy of cuproptosis. In conclusion, cuproptosis is a promising cell death mechanism, and the development of nanomedicines that can precisely control this process may offer an effective anticancer treatment strategy. Combined with an in-depth understanding of the biological pathways of cuproptosis and rational designs of copper-based nanomedicines will provide the potential for effective cancer treatment.

## Data Availability

Data sharing is not applicable to this article as no datasets were generated or analyzed during the current study.
