# Supplementary material for: Copper-Based Nanomedicines for Cuproptosis-Mediated Effective Cancer Treatment
Source: Biomater Res. 2024 Oct 18;28:0094. doi: 10.34133/bmr.0094 (PMC11486892; doi:10.34133/bmr.0094)
Supplement: Supplementary 1 — Figs. S1 to S3 [file bmr.0094.f1.zip › Supplemental Materials.docx]

***<Supplemental Materials>***

**Copper-Based Nanomedicines for Cuproptosis-Mediated Effective Cancer Treatment**

Dahye Noh^1,2^, Hokyung Lee^1,3^, Sangmin Lee^3^, In-Cheol Sun^1^, Hong Yeol Yoon^1,2,*^

^1^Medicinal Materials Research Center, Biomedical Research Institute, Korea Institute of Science and Technology (KIST), Hwarang-ro 14-gil 5, Seongbuk-gu, Seoul 02792, Republic of Korea

^2^Division of Bio-Medical Science &Technology, KIST School, University of Science and Technology (UST), Hwarang-ro14-gil 5, Seongbuk-gu, Seoul 02792, Republic of Korea

^3^Department of Fundamental Pharmaceutical Sciences, College of Pharmacy, Kyung Hee University, 1 Hoegi-dong, Dongdaemun-gu, Seoul, 02447, Republic of Korea

* Corresponding authors.

*E-mail addresses*: seerou@kist.re.kr **(**H.Y. Yoon)


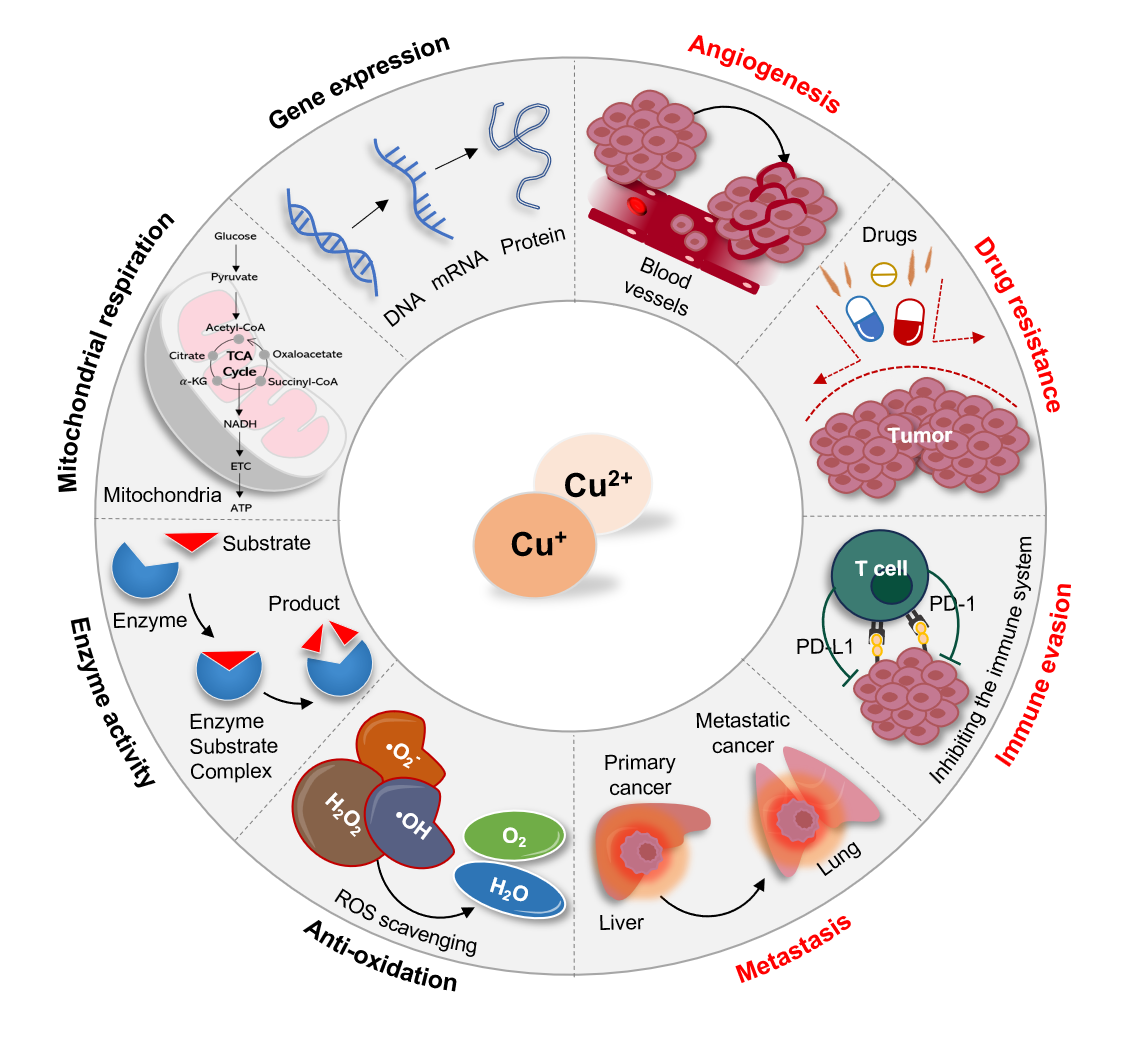


**Fig. S1.** The schematic illustration of the biological roles of copper in healthy cells versus copper ion-overloaded cells, covering gene expression, mitochondrial respiration, enzyme activity, anti-oxidation, metastasis, immune evasion, drug resistance, and angiogenesis.


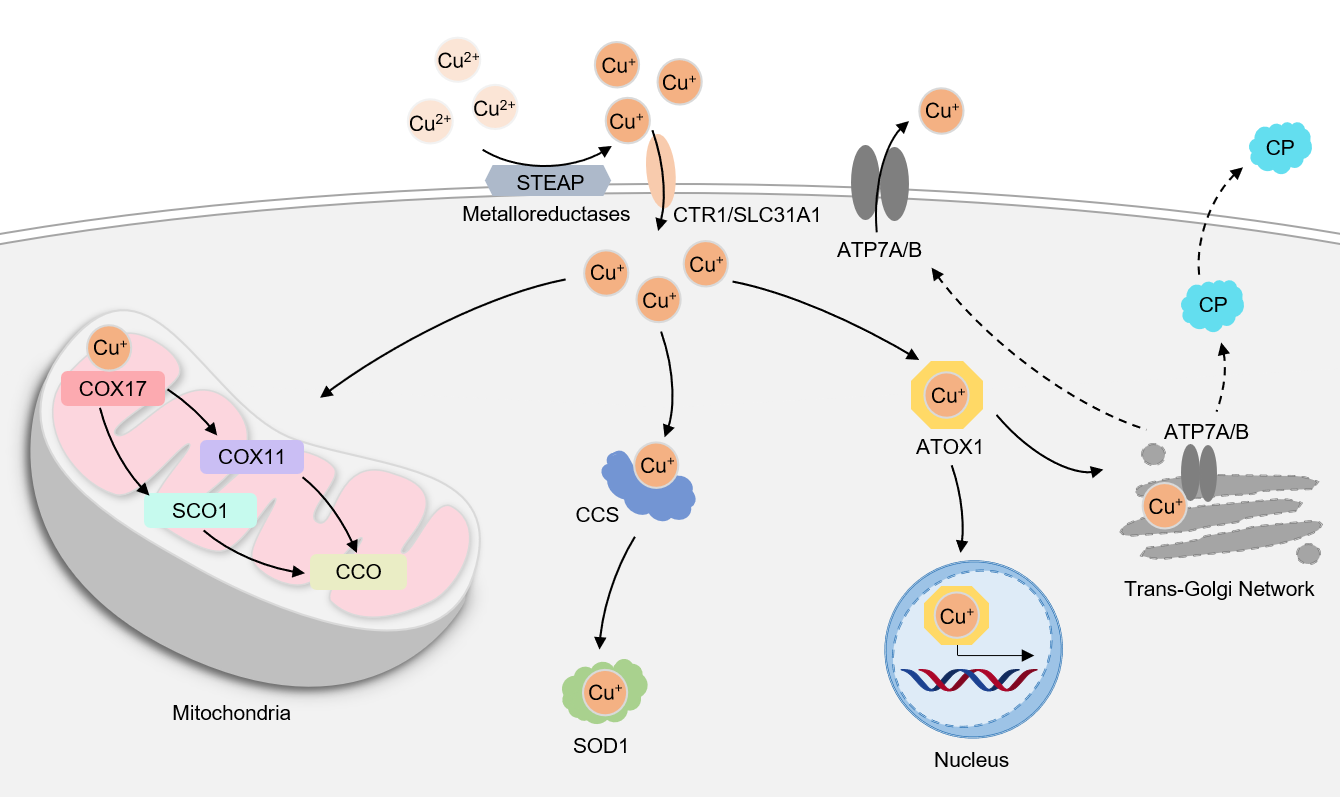


**Fig. S2.** Summary of cellular copper metabolism pathways. Extracellular Cu^2+^ is reduced by the metalloreductase STEAP to Cu^+^, which is internalized into the cell via the copper transporter CTR1 and SLC31A1. Within the cytosol, Cu^+^ is delivered to cytosolic copper chaperones such as CCS and SOD1 before being directed to specific subcellular compartments such as mitochondria, nucleus, and TGN. In the mitochondria, copper ion is involved in the respiratory chain and redox pathways via binding to CCO. In the mitochondrial intermembrane space, COX17 binds to and delivers copper ion to either SCO1 or COX11, which transfers copper ion to the cytochrome oxidase subunit. In the nucleus, copper-bound ATOX1 can bind to transcription factors, influencing gene expression. Finally, in the TGN, copper-ATPase transporters ATP7A and ATP7B transfer copper ion from the cytosol to the TGN lumen, activating copper-dependent enzymes in the secretory pathway. Alternatively, copper ion can be released into the blood stream, binding to CP, amino acids, or albumin for transport. Refer to text for further details.


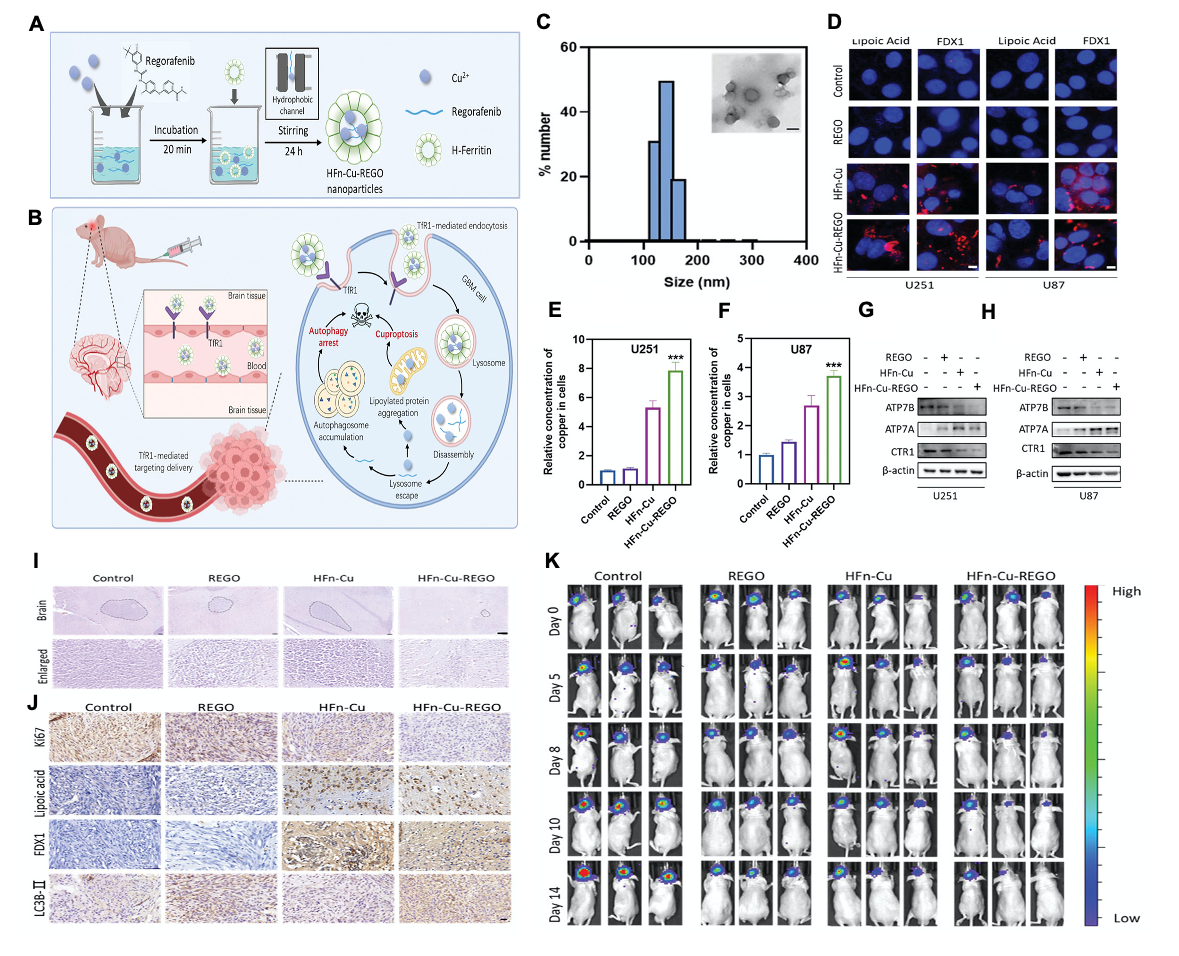


**Fig. S3.** Targeting the brain with HFn-Cu-REGO nanoplatform: precision delivery and modulation of autophagy and cuproptosis in glioblastoma. (A) Schematic illustration of the synthesis process of HFn-Cu-REGO NPs. (B) Therapeutic mechanism illustration of HFn-Cu-REGO NPs in GBM treatment. (C) Dynamic light scattering measurement of the hydrodynamic diameters of HFn-Cu-REGO NPs. (Inset: TEM image of HFn. Scale bars: 20 nm). (D) Immunofluorescence examination of endogenous levels of lipoic acid and FDX1 in GBM cells for 24 h treatment with Regorafenib, HFn-Cu, and HFn-Cu-REGO NPs. Scale bars: 10 µm. (E, F) Copper ion levels in U251 and U87 cell lines. (G, H) Immunoblotting analysis of copper ion transporters in U251 and U87 cell lines. (I) Photographs of HE-stained brain slices following the specified treatments on day 15. (J) Immunohistochemical analysis of Ki67, lipoic acid, FDX1, and LC3B-II levels in brain tumor tissues post-treatment variations on day 15. (K) Bioluminescence imaging of U87-Luc GBM-bearing mice captured on days 0, 5, 8, 10, and 14 post-treatments with PBS, Free regorafenib, HFn-Cu NPs, and HFn-Cu-REGO NPs. Reproduced from [153] with permission from Wiley-VCH, Copyright 2023.
